# For Insulin Signaling Pathways in Flies, Size Matters

**DOI:** 10.1371/journal.pbio.0030320

**Published:** 2005-08-16

Insulin or insulin-like proteins signal developing animals to grow. After a meal, the body creates insulin, allowing an organism to grow and compete with other organisms for available food. When food is scarce, insulin levels remain low. Only small organisms with low metabolic needs will survive the potential famine. Scientists can study how genes involved in insulin signaling affect development by mutating a gene and seeing what happens to the adult. This useful method, called gene knockout, provides insight into the specific relationship between a gene and its physical manifestation, or phenotype. By using the knockout method, scientists can observe how the growth of an organism responds to fluctuations in insulin signaling levels.

In a new study, Alexander Shingleton and colleagues used a temperature-sensitive mutation in an insulin-receptor gene to discover how alterations of insulin signaling in the fruitfly Drosophila affect different stages of fly development. At one stage, the researchers discovered, insulin signaling influences total development time, at another it influences body size, and at a third stage, it influences only organ size.

So when do developing flies need insulin? The researchers found that low insulin signaling during very early development extends total development time. Then the larvae reach their critical size, the watershed moment in insect development when larvae commit to becoming pupae. After critical size, reduced insulin signaling no longer delays development but instead leads to petite flies with petite organs. When the larvae become pupae, however, reduced insulin signaling simply creates smaller organs. Because developmental time, body size, and organ size each display different responses to reduced insulin signaling activity, these features may evolve independently, the authors reasoned. [Fig pbio-0030320-g001]

**Figure pbio-0030320-g001:**
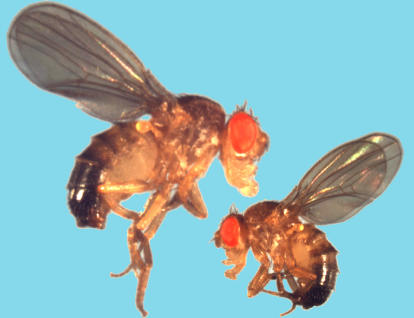
Experiments in the fruitfly show that insulin signaling controls body size, as well as organ size and total development time, by affecting cell growth and proliferation

Shingleton and colleagues used flies with mutant insulin-receptor genes whose protein products are partially inactivated at high temperatures. During different stages of the flies' development, the scientists cranked up the heat (from 17 °C to 24 °C) and watched what happened to their bugs. Using this temperature-sensitive insulin-receptor gene, the researchers found that, besides affecting development time, insulin signaling also plays a role in the differential growth rates of different organs. By tracking three organs on male flies, Shingleton and colleagues discovered that the genitals are less sensitive to reduced insulin signaling than either the wings or the maxillary palps, olfactory components of the mouth. The authors also found that insulin signaling affects cell size and cell number differently. While slightly reduced insulin signaling shrinks cell size, highly reduced insulin signaling lowers cell number without affecting cell size. By incorporating the effects of reduced insulin signaling into the Drosophila development process, the authors constructed a model of Drosophila development that explains the various roles played by the insulin-signaling pathway during development.

Because the new study alters genes during development, it provides the details of when and how a developing animal requires insulin. Future fly studies may reveal why organs have individual responses to insulin signals, what other signaling pathways play a role in development, and how insulin came to influence so many different features of the developing fly at different times.

